# PLX4032 resistance of patient-derived melanoma cells: crucial role of oxidative metabolism

**DOI:** 10.3389/fonc.2023.1210130

**Published:** 2023-07-18

**Authors:** Ombretta Garbarino, Giulia Elda Valenti, Lorenzo Monteleone, Gabriella Pietra, Maria Cristina Mingari, Andrea Benzi, Santina Bruzzone, Silvia Ravera, Riccardo Leardi, Emanuele Farinini, Stefania Vernazza, Melania Grottoli, Barbara Marengo, Cinzia Domenicotti

**Affiliations:** ^1^ Department of Experimental Medicine, General Pathology Section, University of Genoa, Genoa, Italy; ^2^ IRCCS Ospedale Policlinico San Martino, Genova, Italy; ^3^ Department of Experimental Medicine, Biochemistry Section, University of Genoa, Genoa, Italy; ^4^ Department of Experimental Medicine, Human Anatomy Section, University of Genoa, Genoa, Italy; ^5^ Department of Pharmacy, University of Genoa, Genoa, Italy

**Keywords:** melanoma, BRAF, PLX4032, targeted therapy resistance, pyruvate dehydrogenase, glutathione, oxidative metabolism

## Abstract

**Background:**

Malignant melanoma is the most lethal form of skin cancer which shows BRAF mutation in 50% of patients. In this context, the identification of BRAF^V600E^ mutation led to the development of specific inhibitors like PLX4032. Nevertheless, although its initial success, its clinical efficacy is reduced after six-months of therapy leading to cancer relapse due to the onset of drug resistance. Therefore, investigating the mechanisms underlying PLX4032 resistance is fundamental to improve therapy efficacy. In this context, several models of PLX4032 resistance have been developed, but the discrepancy between *in vitro* and *in vivo* results often limits their clinical translation.

**Methods:**

The herein reported model has been realized by treating with PLX4032, for six months, patient-derived BRAF-mutated melanoma cells in order to obtain a reliable model of acquired PLX4032 resistance that could be predictive of patient’s treatment responses. Metabolic analyses were performed by evaluating glucose consumption, ATP synthesis, oxygen consumption rate, P/O ratio, ATP/AMP ratio, lactate release, lactate dehydrogenase activity, NAD^+^/NADH ratio and pyruvate dehydrogenase activity in parental and drug resistant melanoma cells. The intracellular oxidative state was analyzed in terms of reactive oxygen species production, glutathione levels and NADPH/NADP^+^ ratio. In addition, a principal component analysis was conducted in order to identify the variables responsible for the acquisition of targeted therapy resistance.

**Results:**

Collectively, our results demonstrate, for the first time in patient-derived melanoma cells, that the rewiring of oxidative phosphorylation and the maintenance of pyruvate dehydrogenase activity and of high glutathione levels contribute to trigger the onset of PLX4032 resistance.

**Conclusion:**

Therefore, it is possible to hypothesize that inhibitors of glutathione biosynthesis and/or pyruvate dehydrogenase activity could be used in combination with PLX4032 to overcome drug resistance of BRAF-mutated melanoma patients. However, the identification of new adjuvant targets related to drug-induced metabolic reprogramming could be crucial to counteract the failure of targeted therapy in metastatic melanoma.

## 1 Introduction

Malignant melanoma accounts for approximately 2% of all cutaneous malignancies and is responsible for 75% of all skin cancer deaths. The melanoma incidence and death rate have progressively increased during the last 30 years (1), reaching a global incidence of 15-25 per 100,000 individuals with 48,000 deaths according to World Health Organization (2). Current treatment guidelines establish surgical resection as the standard of care for localized and locoregional melanoma (3) and chemotherapy, administered as a single agent or in combination with immunotherapy/biologic drugs, represents the first-line treatment for advanced melanoma. Unfortunately, the acquisition of drug resistance mainly due to the constitutive activation of oncogenes, hampers the effectiveness of therapy in terms of overall survival (4, 5). Indeed, mutations in BRAF (V-raf murine sarcoma viral oncogene homolog B1), a human proto-oncogene member of the RAF (Rapidly growing Fibrosarcoma) family of serine-threonine kinases, have been identified in 50% of malignant melanomas (6), and approximately 40-70% of the cases show a missense mutation, with a substitution of valine with glutamic acid at codon 600 (denoted as V600E) (7). This mutation determines a phosphomimetic conformational change in the activation domain of BRAF leading to a constitutively-activation of MAPK–ERK signalling and a massive increase in the basal kinase activity (8, 9). The identification of oncogenic driver mutations, such as KRAS and BRAF, has accelerated the development of small-molecule inhibitors along the RAS-RAF-MEK-MAPK signaling pathway (10). Currently, PLX4032 (commercially known as Vemurafenib) is approved for the treatment of patients with BRAF^V600E^-mutated metastatic melanomas (11, 12) and, although to a lesser extent, of those with V600K, V600R and V600D mutations (13).

Despite the encouraging results obtained with PLX4032, most BRAF-mutant metastatic melanoma patients become drug-resistant after 6/7 months of therapy, promoting cancer relapse (14).

Interestingly, it has been reported that chemoresistance is associated with an increase in oxidative phosphorylation (OXPHOS) and in mitochondrial biogenesis (10). However, although this metabolic adaptation is particularly observed in PLX4032-resistant BRAF^V600E^ melanoma cells (15), the mechanisms underlying this response are still poorly understood. One of the reasons of this gap is the limited availability of *in vitro* models able to reproduce a reliable condition of therapy resistance occurring *in vivo*. In this regard, it is crucial to carefully evaluate i) the choice of cancer cell line, ii) the strategy of drug administration and iii) the criteria of selecting drug-resistant cancer cells (16).

In this study, we report a model of acquired PLX4032 resistance obtained by chronically treating patient-derived BRAF-mutated melanoma cells with PLX4032. Taking advantage of this model, the role played by the metabolic transition on the acquisition of chemoresistance has been investigated. To our knowledge, this is the first study carried out utilizing a model of drug resistance that is originated from patient-derived melanoma cells and that could be useful to identify new potential targets to hit in order to overcome targeted therapy (TT) resistance.

## 2 Materials and methods

### 2.1 Chemicals

Vemurafenib (PLX4032) was purchased from Selleck Chemicals LLC (Houston, TX, USA) and the stock solutions were prepared using dimethyl sulfoxide (DMSO, Sigma-Aldrich, St. Louis, MO, USA) as a solvent. N-Acetylcysteine (NAC) and dichloroacetate (DCA) were from Sigma-Aldrich and the stock solutions were prepared in sterile water.

### 2.2 Cell lines

All the experiments were performed on MeOV (BRAF^V600E^) and MeTA (BRAF^V600D^) metastatic melanoma cell lines isolated from the biopsies of TT-untreated patients (17). Melanoma cells were maintained in RPMI 1640 medium (Euroclone Spa, Pavia, Italy) supplemented with 10% Fetal Bovine Serum (FBS, Euroclone Spa, Pavia, Italy), 1% L-Glutamine (Euroclone Spa, Pavia, Italy) and 1% Penicillin/Streptomicin (Euroclone Spa, Pavia, Italy) and grown under standard conditions (37°C humidified incubator with 5% CO2).

### 2.3 Selection of cell populations resistant to PLX4032

PLX4032-resistant (PLX-R) and dimethyl sulfoxide-resistant (DMSO-R) MeOV and MeTA cells were selected by treating the whole population of parental cells with increasing concentrations of PLX4032 or DMSO. DMSO-R cells were sensitive to PLX4032 and were used as a comparative model to exclude a toxic effect of DMSO chronic treatment or its potential interference with the effects exerted by PLX4032. Briefly, to obtain drug-resistant cell populations, the cells were seeded twice a week at a density of 1.5 x 10^6^ and treated over six months with PLX4032 (250 nM- 1.5 µM) or with the corresponding volume of DMSO. The authenticity of the selected cells was checked by Short Tandem Repeat (STR) profile analysis performed by the Immunohematology and Transfusion operative unit, IRCCS Ospedale Policlinico San Martino, Genoa. Fifteen highly polymorphic STR loci plus amelogenin (Cell IDTM System, Promega, Milan), were used. Detection of amplified fragments was obtained by ABI PRISM 3100 Genetic Analyzer. Data analysis was performed by GeneMapper^®^ Software version 4.0 (Thermo Fisher Scientific, Waltham, MA, USA).

### 2.4 Treatments

Both chronically-treated melanoma cells were treated for 72 h with increasing concentrations of either PLX4032 (0.1-20 µM) or the corresponding volume of DMSO (0.0025%-0.1% v/v) to verify the efficiency of our long-term-drug-induced resistance protocol. Then, PLX-R and DMSO-R melanoma cells were treated with 1.5 µM PLX4032 or 0.0075% v/v DMSO for 24, 48 or 72 h, according to the experiments. Cell cultures were carefully monitored before and during the experiments to ensure optimal cell density. Notably, samples were discarded if the cell confluence reached > 90%.

In another set of experiments, both DMSO-R cell populations were pre-treated for 2 h with NAC (2mM and 4 mM) or DCA (25 mM or 50 mM) and then exposed for additional 70 h to PLX4032 (1.5 µM and 5 µM).

### 2.5 Cell viability assay

To evaluate cell viability, the CellTiter 96^®^ AQueous One Solution Cell Proliferation Assay (Promega, Madison, WI, USA) was used according to the manufacturer’s instructions. Briefly, cells (10 x 10^3^ cells/well) were seeded into 96-well plates (Corning Incorporated, Corning, NY, USA) and then treated as above described. Finally, cells were incubated with 20 µL of CellTiter and the absorbance at 490 nm was recorded using a microplate reader (EL-808, BIO-TEK Instruments Inc., Winooski, VT, USA).

### 2.6 Cell cycle analysis

To perform cell cycle analysis DMSO-R and PLX-R cells were centrifuged as a mono-disperse cell suspension (about 5 x 10^6^ cells), the supernatant was removed and the cells were re-suspended in cold fixative solution (70% ETOH and 30% PBS) and stored at -20°C for at least 24 h. After one week, the fixative solution was removed by PBS washing and then cells were re-suspended in DNA staining solution (40 μg/ml PI and 0.1mg/ml RNase in PBS) and incubated at room temperature in the dark for 30 min. Finally, all samples were analyzed by the FACScalibur flow cytometer (BD Biosciences).

### 2.7 Glucose consumption

Glucose consumption was evaluated by measuring its concentration in the growth medium using a double beam spectrophotometer (UNICAM UV2, Analytical S.n.c., Langhirano, PR, Italy), by the hexokinase (HK) and glucose 6 phosphate dehydrogenase (G6PD) coupling system, following the reduction of NADP^+^ at 340 nm as previously reported (18).

### 2.8 Oxygen consumption rate (OCR)

OCR was measured by a thermostatically-controlled (37°C) oxygraph apparatus equipped with an amperometric electrode for oxygen (Unisense-Microrespiration, Unisense A/S, Denmark), as previously reported (18).

### 2.9 Evaluation of intracellular ATP and AMP levels

ATP and AMP levels were measured by the enzyme coupling method, following NADP reduction or NADH oxidation, respectively, at 340 nm as previously reported (18).

### 2.10 ATP synthesis

The ATP content was measured using the luciferin/luciferase ATP bioluminescence assay kit CLSII (Roche, Basel, Switzerland) on a Luminometer (Triathler, Bioscan, Washington, DC, USA) (18).

### 2.11 Lactate release and lactate dehydrogenase activity

The concentration of lactate released by melanoma cells in the culture medium was analyzed by spectrophotometric analysis as previously reported (18). Lactate dehydrogenase (LDH) activity was measured following the NADH oxidation at 340 nm. The assay medium contained 100 mM Tris-HCl pH 7.4, 5 mM pyruvate, 40 μM rotenone, and 0.2 mM NADH (19).

### 2.12 NAD(P)/NAD(P)(H)

PLX-R/DMSO-R MeOV and MeTA cells were plated in 24-well plates and treated with 1.5 µM PLX4032 for 24, 48 or 72 h. Cells were then harvested and lysed in 0.1 ml of 0.6 M perchloric acid (PCA; for NAD^+^ and NADP^+^) or of 0.1 M NaOH (for NADH and NADPH) at 4°C. Samples in PCA were neutralized by diluting the extracts in 100 mM sodium phosphate buffer (pH 8); samples in NaOH, were warmed at 72°C for 10 min and were neutralized in 10 mM Tris-HCl, pH 6. NAD(H) content was assessed with an enzyme cyclic assay using alcohol deydrogenase, as previously described (20). NADP(H) content was measured utilizing an alternative enzymatic reaction mixture using glucose-6-phosphate deydrogenase (21). Resazurin reduction to resorufin was monitored at 544 nm excitation, 590 nm emission, using a fluorescence plate reader (Fluostar Optima, BMG Labtechnologies GmbH, Offenburg, Germany). A standard curve was always run in parallel.

### 2.13 Pyruvate dehydrogenase activity

Enzymatic activity of pyruvate dehydrogenase (PDH) was performed using the “Pyruvate dehydrogenase (PDH) Combo (Activity + Profiling) Microplate Assay Kit” provided by Abcam (ab110671, Abcam) and following the manufacturer’s instructions. For each sample, 4 x 10^6^ cells were used (22).

### 2.14 Western blotting analysis

Immunoblots were carried out according to standard methods (23) using rabbit antibody anti-PDH (Cell Signaling Technology Inc., Danvers, MA, USA Upstate, Lake Placid, NY, USA) and anti-rabbit secondary antibodies coupled with horseradish peroxidase (Cell signaling Technologies).

### 2.15 Reactive oxygen species production

Detection of Reactive Oxygen Species (ROS) levels was evaluated by incubating cells with 5 µM 2'-7' dichlorofluoresceindiacetate (DCFH-DA; Sigma-Aldrich) as previously reported (18).

### 2.16 Total glutathione levels

Total glutathione (GSH) content was analyzed as previously reported (18).

### 2.17 Principal component analyses

Principal component analysis (PCA) was performed as previously described (24) by using the CAT software. (R. Leardi, C. Melzi, G. Polotti, CAT (Chemometric Agile Tool), freely downloadable from http://gruppochemiometria.it/index.php/software).

### 2.18 Statistical analysis

Results were expressed as mean ± SEM from at least four independent experiments. The statistical significance of parametric differences among the sets of experimental data was evaluated by one-way ANOVA and Dunnett’s test for multiple comparisons.

## 3 Results

### 3.1 Chronic exposure of BRAF-mutated melanoma cells to PLX4032 leads to the selection of PLX4032-resistant cell lines

Since BRAF-mutated patients treated with PLX4032 have been reported to develop resistance after 6 months, we decided to investigate the effects induced by PLX4032 chronic treatment of BRAF^V600E^ MeOV and BRAF^V600D^ MeTA that are human metastatic melanoma cell lines derived from two patients. Therefore, both melanoma cell lines were treated for 6 months with increasing concentrations of PLX4032 (1 nM-1.5 μM) and then maintained in culture with the same drug at the highest concentration. Notably, in all experiments, PLX4032 chronically-treated cells were compared to the DMSO chronically-treated ones.

**Figure 1:**
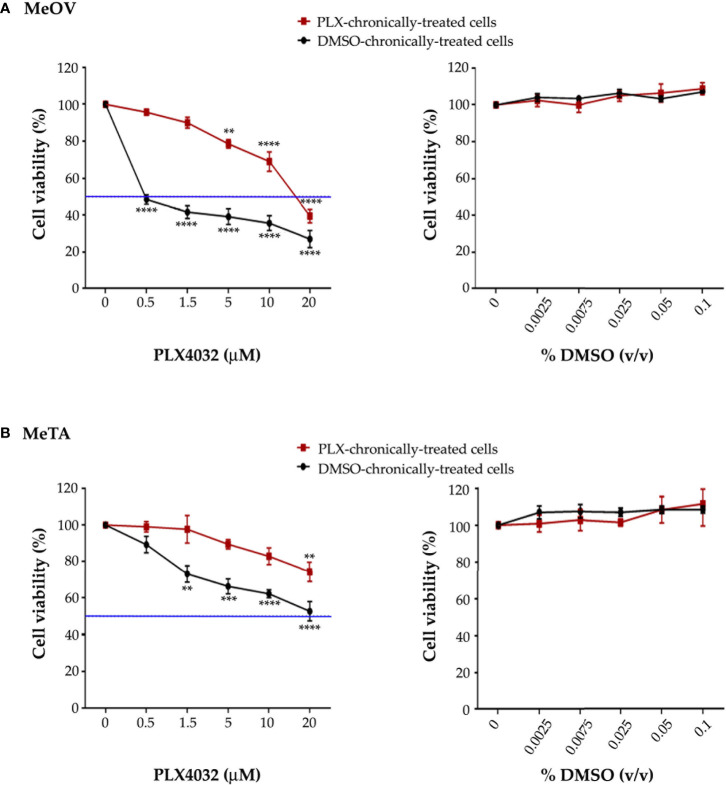
Chronic exposure of MeOV **(A)** and MeTA **(B)** melanoma cells to PLX4032 or DMSO is able to select a resistant phenotype. PLX4032 and DMSO chronically-treated MeOV and MeTA cells were exposed for 72 h to increasing concentrations (0.1-20 µM) of PLX4032 (left panels) or DMSO (0.0025-0.1% v/v) (right panels) and cell viability was analyzed by MTT assay. The blue line is drawn at 50% cell viability. Graphs summarize quantitative data of the means ± S.E.M. of four independent experiments. **p<0.01 vs. untreated cells; ***p<0.001 vs. untreated cells; ****p<0.0001 vs. untreated cells.

The efficiency of the long-term-drug-induced resistance protocol above described was tested by analyzing the viability of MeOV and MeTA cells treated for 72 h with increasing (0.1-20 µM) PLX4032 or (0.0025%-0.1% v/v) DMSO concentrations. As shown in **Figure 1**, PLX4032 induced dose-dependent cytotoxic effects in DMSO chronically-treated cells selected from either MeOV (panel A, left) and MeTA (panel B, left). However, PLX4032 was more cytotoxic for MeOV cells, having an IC_50_ of 0.5 μM (**Figure 1A**, left panel, green line), while for MeTA cells the IC_50_ was 18.7 μM (**Figure 1B**, left panel, green line). Moreover, analyzing the effects of the same treatments on PLX4032 chronically-treated cells, 5 μM PLX4032 was found to reduce the viability of MeOV cells by 22%, while the highest dose (20 μM) of the drug induced 60% of cell death reaching an IC_50_ of 16.2 μM (**Figure 1A**, left panel). Instead, the viability of MeTA cells was reduced by 20% only after the treatment with 20 μM PLX4032 and the IC_50_ was 37.2 μM (**Figure 1B**, left panel).

Notably, all concentrations of DMSO, corresponding to the volume of the solvent employed to dissolve PLX4032, did not affect *per se* the survival of all melanoma cells (**Figures 1A, B**, right panels).

Since the ratio of PLX4032 IC_50_ values in PLX4032 and DMSO chronically-treated cells was 32.4 (MeOV) and 2 (MeTA), both PLX4032-selected populations can be considered resistant to the drug (PLX-R).

The identity of melanoma cell lines was verified and certified, comparing parental cell line profile to the two profiles of resistant cell lines (PLX-R and DMSO-R). As shown in **Table 1**, the results demonstrated an identical profile of Short Tandem Repeat (STR) sequence loci in all melanoma cell lines, certifying the authenticity of the selected cells and demonstrating lack of contamination from other cell lines and microbes.

**Table 1:** Short Tandem Repeat sequence loci in parental, DMSO-R and PLX-R melanoma cells.

	Cell name	D 5S818	D 13S317	D 7S820	D 16S539	VWA	TH01	AM	TPOX	CSF 1PO	D 21S11	D 3S1358	D 18S51	Penta E	Penta D	D 8S1179	FGA
**MeOV**	**Parental**	12.13	11	7.9	11	15.17	6	X	8	10	33.2	15	12	12	9.12	13	21.22
	**DMSO-R**	12.13	11	7.9	11	15.17	6	X	8	10	33.2	15	12	12	9.12	13	21.22
	**PLX-R**	12.13	11	7.9	11	15.17	6	X	8	10	33.2	15	12	12	9.12	13	21.22
**MeTA**	**Parental**	11.12	12.13	11	11	17.18	8.9.3	X	8.10	12.13	29.30	17	18	7.10	9	11.15	21.22
	**DMSO-R**	11.12	12.13	11	11	17.18	8.9.3	X	8.10	12.13	29.30	17	18	7	9	11.15	21.22
	**PLX-R**	11.12	12.13	11	11	17.18	8.9.3	X	8.10	12.13	29.30	17	18	7.10	9	11.15	21.22

Furthermore, since acute PLX4032 exposure markedly decreased the viability of both DMSO-R cell populations, the effect of the drug on the cell cycle was investigated. As shown in **Table 2**, the percentage of MeOV-DMSO-R cells in G0/G1 phase increased by 30, 20 and 13% compared to untreated cells, after 24, 48 and 72 h of treatment with PLX4032, respectively. This effect was accompanied by a concomitant decrease in the percentage of cells in S and G2/M phase. A similar trend in cell cycle phases distribution was observed in MeTA-DMSO-R cells but with a reduced effect in respect to MeOV cells. Interestingly, in both PLX-R cell populations, acute drug exposure did not affect the distribution of cells in cell cycle phases that was comparable to that observed in untreated ones.

**Table 2:** Cell cycle analysis.

	MeOV			MeTA		
	G0/G1 (%)	S (%)	G2/M (%)	G0/G1 (%)	S (%)	G2/M (%)
**DMSO-R untreated (24 h)**	57.667 ± 3.18	13.333 ± 1.202	25.33 ± 2.18	69.8 ± 3.105	17.43 ± 1.567	10 ± 3.629
**DMSO-R + 1.5 μM PLX (24 h)**	89.333 ± 0.667**^^^^^^**	2.667 ± 0.333**^^^^^^**	6.667 ± 0.333**^^^^^**	84.867 ± 4.699**^^^**	5.967 ± 2.53**^^^**	7.97 ± 2.310
**DMSO-R untreated (48 h)**	69.333 ± 3.48	12 ± 1.155	15.67 ± 1.764	70.2 ± 5.077	17 ± 1.528	10.2 ± 3.617
**DMSO-R + 1.5 μM PLX (48 h)**	89.333 ± 1.202^**^	2 ± 0.0^****^	7.33 ± 0.882^*^	86.733 ± 2.339^*^	4.967 ± 0.033^*^	6.5 ± 1.607
**DMSO-R untreated (72 h)**	72 ± 4.359	10.667 ± 2.028	14.67 ± 1.856	64.467 ± 5.002	19.4 ± 3.124	13 ± 2.537
**DMSO-R + 1.5 μM PLX (72 h)**	85.333 ± 3.48^*^	3 ± 0.577^****^	10.33 ± 2.404	82.867 ± 3.994^*^	7.33 ± 2.963^*^	7.47 ± 1.444
**PLX-R untreated (24 h)**	49.333 ± 5.548	9.333 ± 0.882	31.67 ± 3.180	72.2 ± 2.996	17 ± 3.606	9.27 ± 1.392
**PLX-R + 1.5 μM PLX (24 h)**	61.333 ± 5.783	6.667 ± 0.333	25.33 ± 4.485	79.567 ± 4.71	12.8 ± 3.8	5.33 ± 0.333
**PLX-R untreated (48 h)**	50.667 ± 2.963^*^	12.667 ± 0.333	27.33 ± 1.856^*^	74 ± 3.055	17.33 ± 3.844	8 ± 2.646
**PLX-R + 1.5 μM PLX (48 h)**	53 ± 4.509	10.667 ± 0.667	27.67 ± 3.180	77.333 ± 1.202	14.8 ± 3.252	7.5 ± 2.021
**PLX-R untreated (72 h)**	51.667 ± 2.404^**^	11.333 ± 0.882	28.33 ± 1.856^*^	70.333 ± 4.256	18.33 ± 3.283	10.3 ± 2.333
**PLX-R + 1.5 μM PLX (72 h)**	53 ± 4.359	10.667 ± 0.882	28.67 ± 4.256	72.9 ± 1.646	16.33 ± 2.186	9.7 ± 1.350

^^^^*p*<0.0001 vs untreated DMSO-R 24 h; ^^^*p*<0.001 vs untreated DMSO-R 24 h; ^*p*<0.05 vs untreated DMSO-R 24 h; ****p<0.0001 vs untreated DMSO-R 48 h; **p<0.01 vs untreated DMSO-R 48 h; *p<0.05 vs untreated DMSO-R 48 h; **p<0.01 vs untreated DMSO-R 72 h; *p<0.05 vs untreated DMSO-R 72 h.

Cell cycle was evaluated in DMSO- and PLX-R melanoma cells exposed to 1.5 µM PLX4032 PLX or to DMSO for 24, 48 and 72 h.

### 3.2 The acquisition of PLX4032 resistance is accompanied by the ability of melanoma cells to maintain an efficient OXPHOS metabolism

Since the metabolic plasticity of melanoma under drug-induced stress conditions has been demonstrated to confer a survival advantage (25), the metabolic profile of all melanoma cell lines has been investigated.

**Figure 2:**
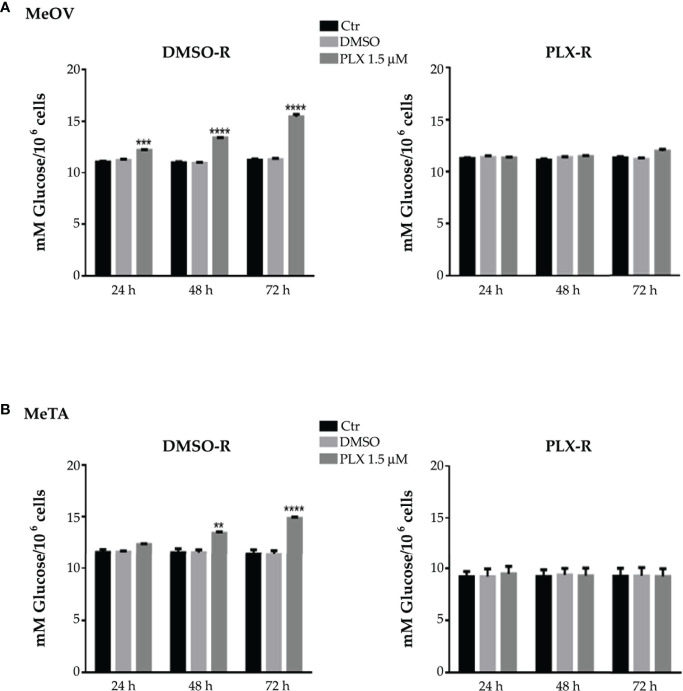
Analysis of glucose consumption in DMSO-R (left panels) and PLX-R (right panels) MeOV **(A)** and MeTA **(B)** cells exposed to 1.5 µM PLX4032 or to DMSO for 24, 48 and 72 h. Results were reported as mM glucose/10^6^ cells. Histograms summarize quantitative data of the means ± S.E.M. of four independent experiments. **p<0.01 vs. untreated cells (Ctr); ***p<0.001 vs. untreated cells (Ctr); ****p<0.0001 vs. untreated cells (Ctr).

As shown in **Figure 2** (A and B, left panels), 1.5 μM PLX4032 increased glucose consumption in both DMSO-R cell populations in a time-dependent manner. However, this effect was already evident at 24 h in DMSO-R MeOV (**Figure 2A**, left panel), while it was observed only at 48 h in DMSO-R MeTA (**Figure 2B**, left panel). Instead, PLX4032 treatment did not alter glucose consumption in both PLX-R cells (**Figures 2A, B**, right panels). Furthermore, DMSO did not induce changes in glucose consumption in all melanoma cell populations (**Figures 2A, B**).

Since cancer cells consume high levels of glucose to satisfy their energetic need in terms of ATP production through OXPHOS or aerobic glycolysis, the two metabolic pathways were characterized. Initially, ATP synthesis and the oxygen consumption rate (OCR) were analyzed under all treatment conditions, using pyruvate plus malate (P/M) or succinate (succ) to stimulate the pathways led by Complex I or Complex II, respectively.

**Figure 3:**
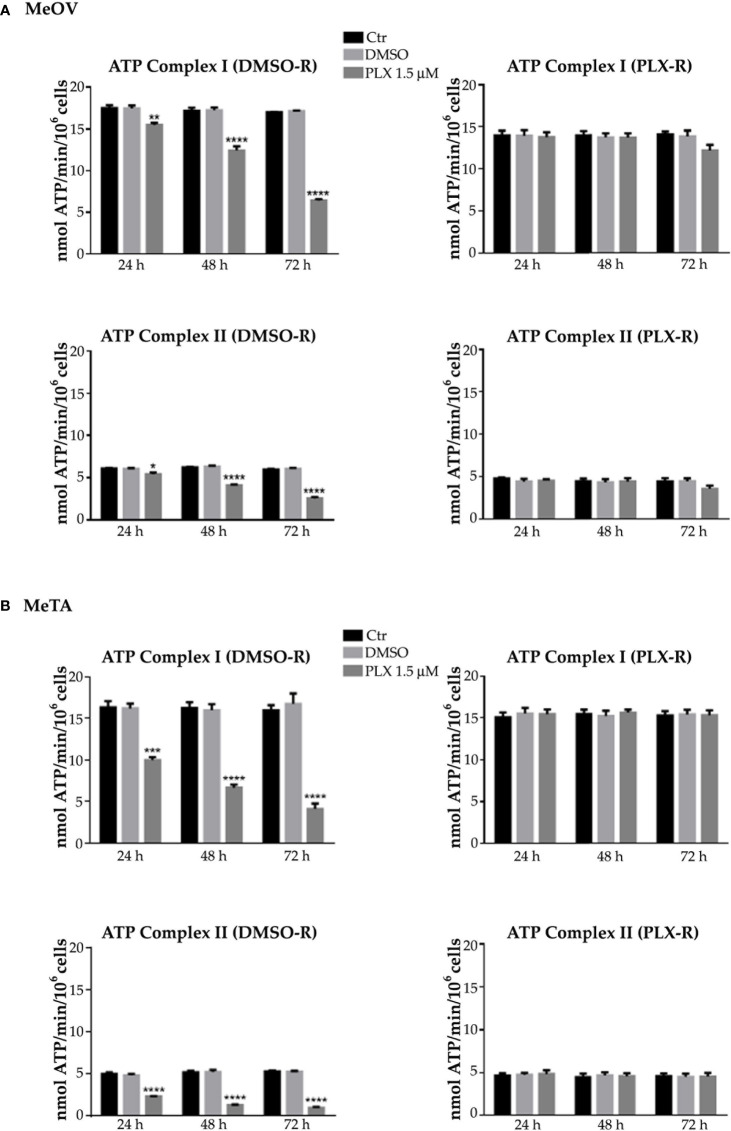
Analysis of ATP synthesis using pyruvate plus malate (P/M, Complex I, upper panels) or succinate (succ, Complex II, lower panels) in DMSO-R (left panels) and PLX-R (right panels) MeOV **(A)** and MeTA **(B)** cells exposed to 1.5 µM PLX4032 or to DMSO for 24, 48 and 72 h. Results were reported as nmol ATP/min/10^6^ cells. Histograms summarize quantitative data of the means ± S.E.M. of four independent experiments. *p<0.1 vs. untreated cells (Ctr); **p<0.01 vs. untreated cells (Ctr); ***p<0.001 vs. untreated cells (Ctr); ****p<0.0001 vs. untreated cells (Ctr).

**Figure 4:**
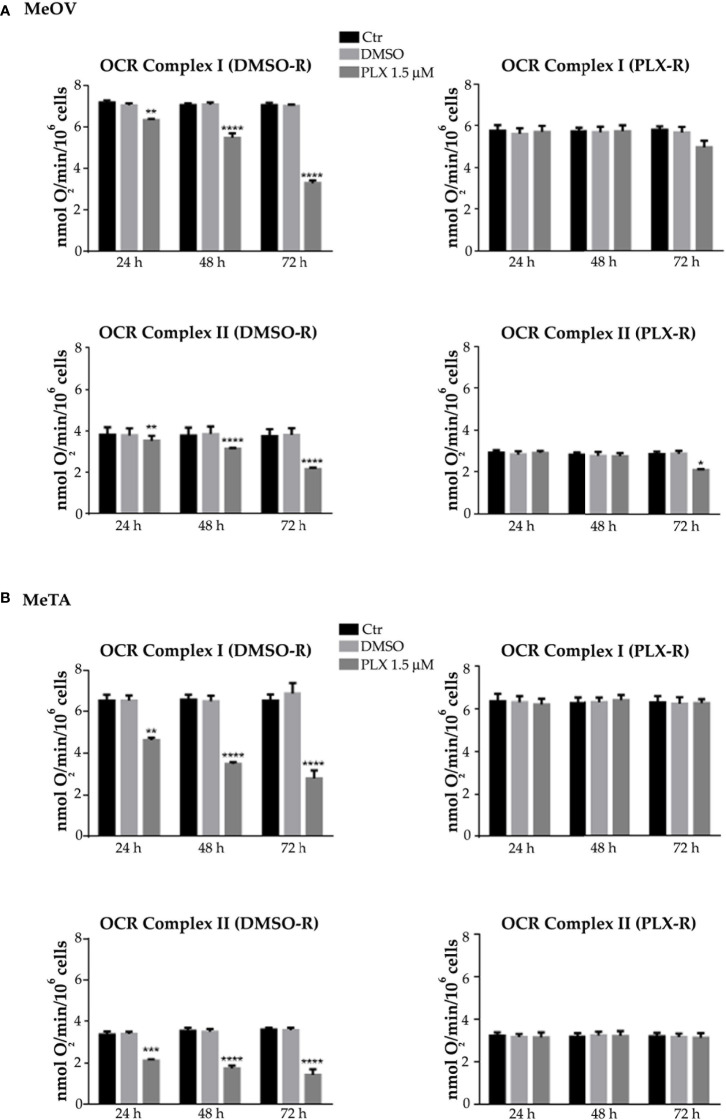
Analysis of oxygen consumption rate (OCR) using pyruvate plus malate (P/M, Complex I, upper panels) or succinate (succ, Complex II, lower panels) in DMSO-R (left panels) and PLX-R (right panels) MeOV **(A)** and MeTA **(B)** in cells exposed to 1.5 µM PLX4032 or to DMSO for 24, 48 and 72 h. Results were reported as nmol O_2_/min/10^6^ cells. Histograms summarize quantitative data of the means ± S.E.M. of four independent experiments. **p<0.01 vs. untreated cells (Ctr); ***p<0.001 vs. untreated cells (Ctr); ****p<0.0001 vs. untreated cells (Ctr).

The values of ATP synthesis (**Figure 3A**, left panels) and the OCR (**Figure 4A**, left panels) of DMSO-R MeOV cells were 25% higher than those measured in PLX-R ones (**Figures 3A**, **4A**, right panels) and were not influenced by DMSO treatment (**Figures 3A**, **4A**). Instead, both parameters were comparable in DMSO-R and PLX-R MeTA cells (**Figures 3B**, **4B**) and DMSO exposure did not modify the trend (**Figures 3B**, **4B**).

As shown in **Figure 3** and **4** (left panels), 1.5 μM PLX reduced ATP synthesis and OCR by both DMSO-R cell populations, in a time-dependent manner and more markedly in MeTA cells. In detail, in DMSO-R MeOV cells, PLX at the longest time of treatment (72 h) reduced P/M-induced ATP synthesis by 47% and succ-induced ATP synthesis by 58% (**Figure 3A**, left panels) while OCR-linked to Complex I and Complex II pathways were decreased by 57% and 42%, respectively (**Figure 4A**, left panels). In DMSO-R MeTA cells, 72 h PLX exposure reduced P/M-induced ATP synthesis by 75% and succ-induced ATP synthesis by 82% (**Figure 3B**, left panels), while OCR-linked Complex I and Complex II pathways were decreased by 57% and 55%, respectively (**Figure 4B**, left panels).

Notably, no changes in ATP synthesis and OCR were recorded in both PLX-R melanoma cells under the same conditions of treatment (**Figures 3**, **4**, right panels).

Moreover, the efficiency of the OXPHOS was evaluated by calculating the P/O ratio expressed as the ratio between the concentration of the synthesized ATP and the amount of consumed oxygen in the presence of respiring substrates and ADP. Under physiological conditions, when the oxygen consumption is completely devoted to the energy production, P/O ratio should be around 2.5 and 1.5 after pyruvate/malate or succinate addition, respectively (26). As shown in **Table 3**, in untreated (Ctr) and DMSO-treated DMSO-R melanoma cells, the P/O ratio was comparable to the physiological level (26) during all time-range analyzed (24, 48 and 72 h). However, in DMSO-R MeOV cells, 24 h PLX4032 exposure did not alter P/O ratio that reached a 18.7% reduction, associable with a partial OXPHOS uncoupling, after 72 h PLX4032 treatment. In DMSO-R MeTA cells, PLX4032 reduced P/O ratio by 23% and 40% after 48 and 72 h respectively, leading to OXPHOS uncoupling. Instead, in both PLX-R melanoma cells, PLX4032 or DMSO exposure did not induce changes in P/O ratio which was always maintained at the physiological level (**Table 3**).

**Table 3:** P/O ratio.

		24 hrs			48 hrs			72 hrs		
		Ctr	DMSO	PLX	Ctr	DMSO	PLX	Ctr	DMSO	PLX
**MeOV DMSO-R**	**Compl. I**	2.43 ± 0.03	2.47 ± 0.03	2.44 ± 0.10	2.43 ± 0.06	2.43 ± 0.05	2.26 ± 0.01*	2.41 ± 0.06	2.44 ± 0.02	1.96 ± 0.21^*^
	**Compl. II**	1.63 ± 0.3	1.63 ± 0.26	1.55 ± 0.22	1.68 ± 0.3	1.68 ± 0.3	1.32 ± 0.02*	1.62 ± 0.3	1.62 ± 0.3	1.18 ± 0.09^*^
**MeOV PLX-R**	**Compl. I**	2.43 ± 0.06	2.47 ± 0,14	2.41 ± 0.11	2.43 ± 0.09	2.41 ± 0.05	2.38 ± 0.08	2.42 ± 0.05	2.44 ± 0.01	2,46 ± 0.06
	**Compl. II**	1.65 ± 0.09	1.57 ± 0.14	1.57 ± 0.14	1.58 ± 0.10	1.55 ± 0.11	1.6 ± 0.14	1.55 ± 0.14	1.55 ± 0.15	1.69 ± 0.32
**MeTA DMSO-R**	**Compl. I**	2.50 ± 0.05	2.48 ± 0.02	2.15 ± 0.07**^^^**	2.47 ± 0.04	2.45 ± 0.09	1.91 ± 0.20*	2.44 ± 0.03	2.44 ± 0.02	1.45 ± 0.10^*^
	**Compl. II**	1.49 ± 0.07	1.43 ± 0.03	1.11 ± 0.10**^^^**	1.47 ± 0.02	1.50 ± 0.02	0.78 ± 0.13*	1.47 ± 0.02	1.47 ± 0.06	0.73 ± 0.15^*^
**MeTA PLX-R**	**Compl. I**	2.37 ± 0.07	2.46 ± 0.02	2.48 ± 0.05	2.47 ± 0.04	2.41 ± 0.09	2.44 ± 0.06	2.43 ± 0.07	2.47 ± 0.07	2.44 ± 0.05
	**Compl. II**	1.45 ± 0.03	1.50 ± 0.08	1.54 ± 0.04	1.41 ± 0.17	1.45 ± 0.08	1.44 ± 0.13	1.44 ± 0.11	1.44 ± 0.16	1.45 ± 0.11

^*p*<0.05 vs untreated DMSO-R 24 h; *p<0.05 vs untreated DMSO-R 48 h; ^*^ *p*<0.05 vs untreated DMSO-R 72 h

The P/O ratio was evaluated in DMSO- and PLX-R melanoma cells exposed to 1.5 µM PLX4032 (PLX) or to DMSO for 24, 48 and 72 h.

**Figure 5:**
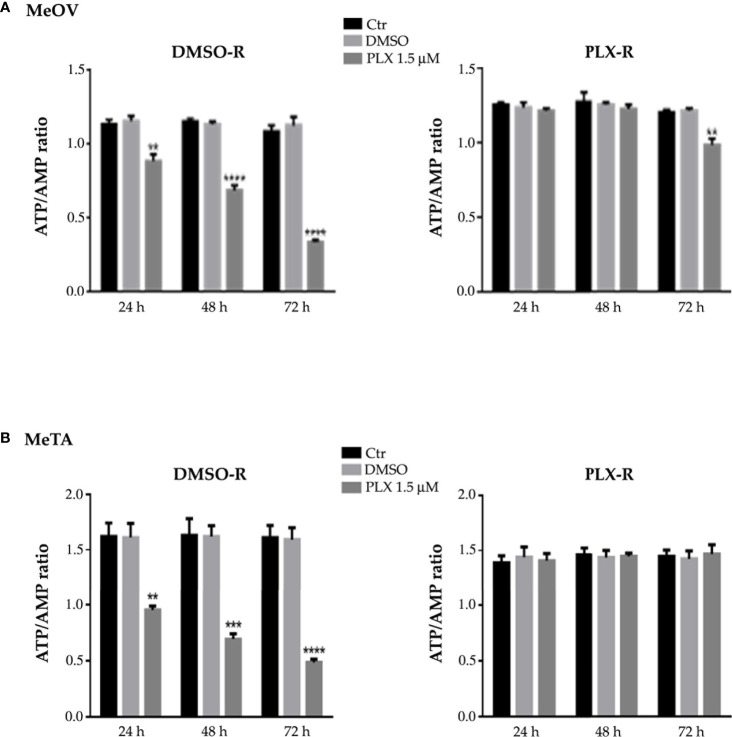
Analysis of ATP and AMP content in DMSO-R (left panels) and PLX-R (right panels) MeOV **(A)** and MeTA **(B)** melanoma cells, exposed to 1.5 µM PLX4032 or to DMSO for 24, 48 and 72 h. Histograms summarize quantitative data of the means ± S.E.M. of four independent experiments. **p<0.01 vs. untreated cells (Ctr); ***p<0.001 vs. untreated cells (Ctr); ****p<0.0001 vs. untreated cells (Ctr).

In addition, as shown in **Figure 5**, untreated DMSO-R and PLX-R melanoma cells had comparable ATP/AMP ratio, at all three analyzed timepoints (24, 48 and 72 h). DMSO exposure did not influence the ATP/AMP ratio in DMSO-R and PLX-R cells. Following acute PLX4032 treatment DMSO-R (MeOV and MeTA) cells displayed a time-dependent decrease in intracellular ATP and an increase in AMP levels (**Supplementary Table 1**) leading to a 70% reduction of ATP/AMP ratio after 72 h in comparison to untreated cells (**Figure 5**, left panel). Instead, acute PLX4032 treatment of both PLX-R cells was able to keep constant the ATP/AMP ratio since it did not modify ATP and AMP levels (**Figure 5**, **Supplementary Table 1**). Only 72 h PLX4032 exposure reduced the ratio by 20% in PLX-R MeOV cells (**Figure 5A**, right panel).

### 3.3 PLX4032 resistant melanoma cells are able to maintain an increased PDH activity and high levels of GSH

In order to better investigate the energetic metabolism, the lactate release and LDH activity were analyzed.

**Figure 6:**
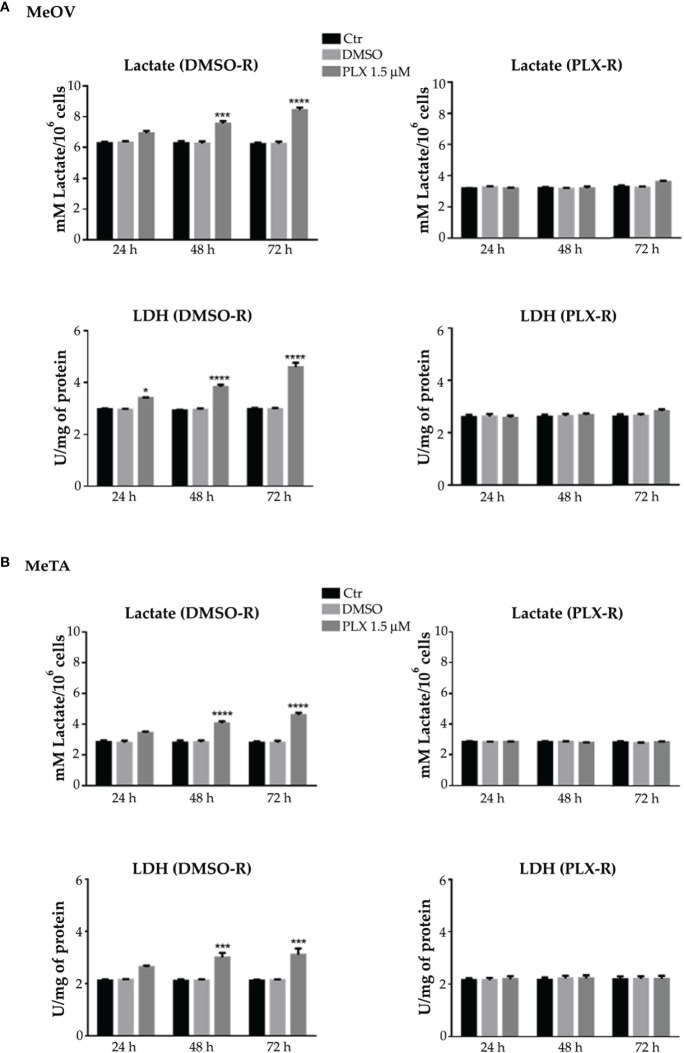
Analysis of extracellular lactate concentration and lactate dehydrogenase activity (LDH) in DMSO-R (left panels) and PLX4032-R (right panels) MeOV **(A)** and MeTA **(B)** exposed to 1.5 µM PLX4032 or DMSO for 24, 48 and 72 h. Results were reported as mM lactate/10^6^ cells for lactate release and as U/mg (lactate μmol/min/mg of total protein) for LDH activity. Histogram summarizes quantitative data of the means ± S.E.M. of four independent experiments. *p<0.1 vs. untreated cells (Ctr); ***p<0.001 vs. untreated cells (Ctr); ****p<0.0001 vs. untreated cells (Ctr).

In PLX4032-treated DMSO-R melanoma cells the lactate release and LDH activity increased in a time-dependent manner (**Figures 6A, B**, left panels) instead, in PLX4032-treated PLX-R cells no statistical increase was observed (**Figures 6A, B**, right panels). Notably, DMSO did not induce changes in all cell populations (**Figures 6A, B**).

**Figure 7:**
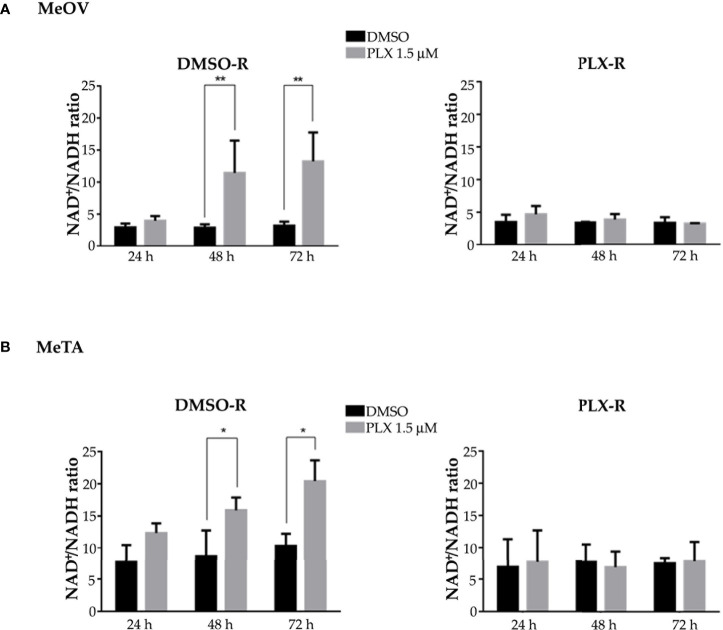
Evaluation of NAD^+^/NADH ratio in DMSO-R (left panels) and PLX-R (right panels) Me-OV **(A)** and MeTA **(B)** melanoma cells, exposed to 1.5 µM PLX4032 or to DMSO for 24, 48 and 72 h. Histograms summarize quantitative data of the means ± S.E.M. of four independent experiments. *p<0.1 vs. untreated cells (Ctr); **p<0.01 vs. untreated cells (Ctr).

Moreover, NADH and NAD^+^ levels were analyzed and the NAD^+^/NADH ratio was calculated. As reported in **Figure 7**, 48 and 72 h PLX4032 treatment increased the ratio by approximately 185% and 230% in DMSO-R MeOV cells respectively (A, left panel) and by 30% and 65% in DMSO-R MeTA cells (B, left panel). Instead, in both PLX4032-treated PLX-R cells the NAD^+^/NADH ratio showed no significant changes (**Figures 7A, B**, right panels).

**Figure 8:**
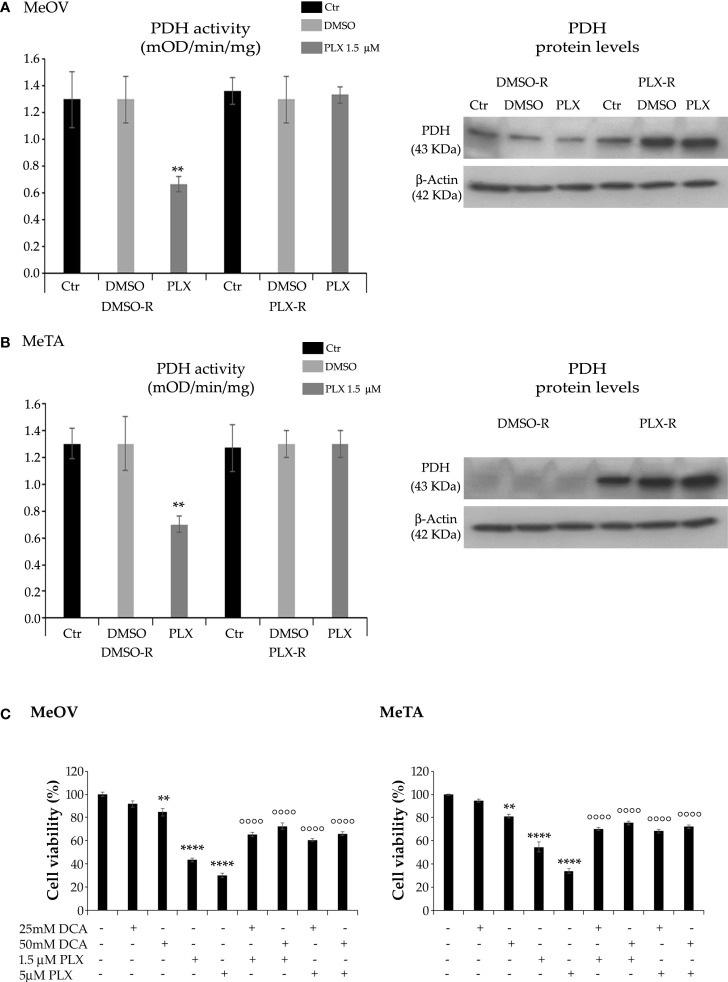
Analysis of PDH activity (left panels) and protein levels (right panels) in DMSO-R and PLX4032-R MeOV **(A)** and MeTA **(B)** melanoma cells treated with 1.5 µM PLX4032 or with DMSO for 72 h. Results were expressed as mOD/min/mg protein. Immunoblots shown are representative of three independent experiments. β-Actin is the internal loading control. **(C)** DCA pre-treatment of DMSO-R cells reduces the cytotoxic effect of PLX4032. Cell viability was determined by MTT assays in MeOV (left panel) and MeTA (right panel) cells pre-treated for 2 h with 25mM or 50 mM DCA and then exposed for additional 70 h to 1.5 μM or 5 μM PLX4032. Histograms summarize quantitative data of the means ± S.E.M. of four independent experiments. **p<0.01 vs. untreated cells (Ctr); ****p<0.0001 vs. untreated cells (Ctr); °°°° *p*<0.0001 vs. PLX-4032-treated cells.

Taking into consideration the different energetic metabolism of DMSO-R and PLX-R melanoma cells, the activity of PDH, the enzyme able to convert pyruvate to acetyl-CoA (27), was analyzed. Accordingly, to NAD^+^/NADH ratio, PDH activity was reduced by 50% in both DMSO-R cells after 72 h PLX4032 exposure while it was maintained constant in treated and untreated PLX-R cells (**Figures 8A, B**). Analysis of PDH protein expression shows that both PLX-R cell populations had higher levels of the enzyme compared to DMSO-R ones (**Figures 8A, B**). Moreover, to better investigate the role of PDH in PLX4032 resistance, both DMSO-R cells were pre-treated for 2 h with DCA, an inhibitor of pyruvate dehydrogenase kinase thus representing a PDH activator, and then exposed to PLX4032 for additional 70 h. Interestingly, the pre-treatment with 25 mM or 50 mM DCA increased the cell viability of both DMSO-R cells by 50% and 68%, respectively, in comparison with 1.5 μM PLX-treated ones (**Figure 8C**) and by 100% and 120% respectively, compared to 5 μM PLX-treated ones (**Figure 8C**).

**Figure 9:**
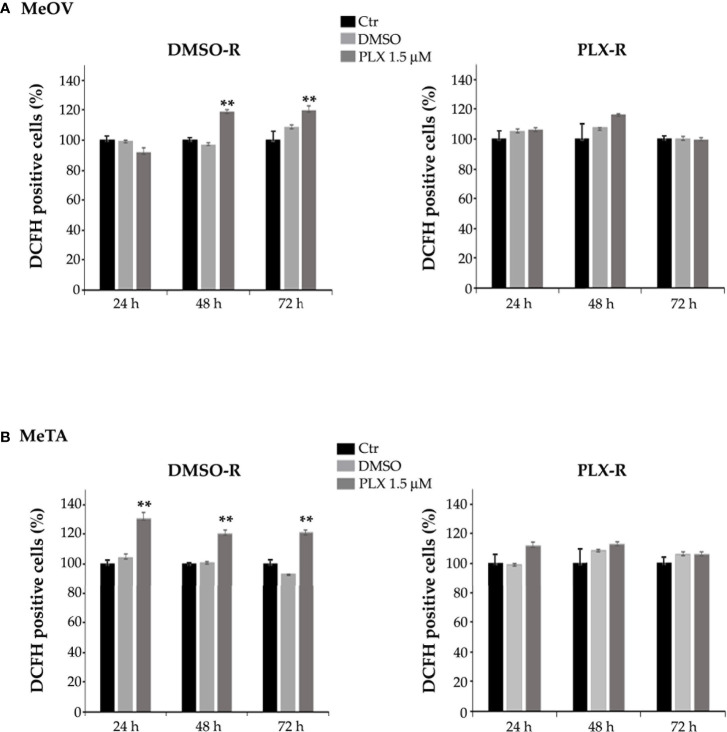
Analysis of ROS production in DMSO-R (left panels) and PLX4032-R (right panels) MeOV **(A)** and MeTA **(B)** melanoma cells, exposed to 1.5 µM PLX4032 or to DMSO for 24, 48 and 72 h. Histograms summarize quantitative data of the means ± S.E.M. of four independent experiments. **p<0.01 vs. untreated cells (Ctr).

Furthermore, to characterize the different metabolic profile, the redox state was investigated in terms of ROS production (28, 29) and GSH levels under all treatment conditions. As shown in **Figure 9**, PLX4032 treatment enhanced ROS levels in both DMSO-R cells (**Figure 9B**, left panel). Instead, no change in ROS production was observed in PLX-R cells under the same treatment conditions (**Figures 9A, B**, right panels).

**Figure 10:**
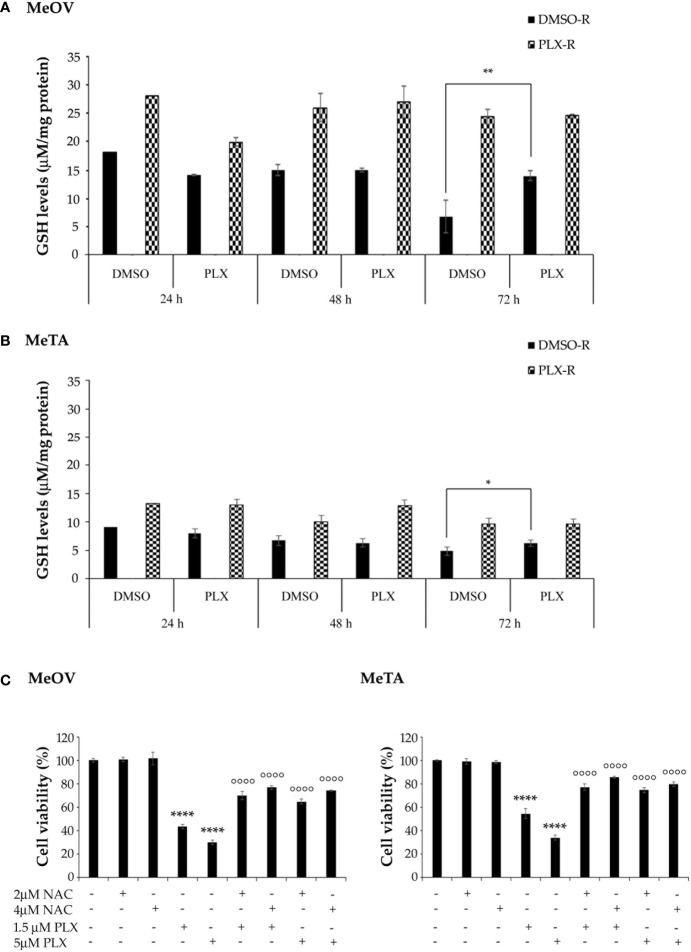
Analysis of total GSH levels in DMSO-R and PLX4032-R MeOV **(A)** and MeTA **(B)** melanoma cells exposed to 1.5 µM PLX4032 or DMSO for 24, 48 and 72 h. Results were reported as μM/μg protein. **(C)** NAC pre-treatment of DMSO-R cells reduces the cytotoxic effect of PLX4032. Cell viability was determined by MTT assays in MeOV (left panel) and MeTA (right panel) cells pre-treated for 2 h with 2 μM or 4 μM NAC and then exposed for additional 70 h to 1.5 μM or 5 μM PLX4032. Histograms summarize quantitative data of the means ± S.E.M. of four independent experiments. *p<0.01 vs. untreated cells (Ctr); **p<0.01 vs. untreated cells (Ctr); ****p<0.0001 vs. untreated cells (Ctr); °°°° *p*<0.0001 vs. PLX-4032-treated cells.

With regard to GSH levels, as shown in **Figure 10**, both PLX-R cells displayed a markedly higher content of GSH in respect to DMSO-R cells (*p*<0.01). Interestingly, under the conditions characterized by the enhanced ROS production, a reduction of GSH levels was not observed. Instead, a significant increase of GSH levels was detected in DMSO-R Me-OVcells after 72 h PLX4032 exposure (**Figure 10A**). Moreover, to confirm the role of GSH in drug resistance, both DMSO-R cells were pre-treated for 2 h with 2 mM or 4 mM NAC, an aminothiol and synthetic precursor of intracellular cysteine, and then exposed to PLX4032 for additional 70 h. The pre-treatment with 2 μM or 4 μM NAC increased the cell viability of both DMSO-R cells by 63% and 79%, respectively, in respect to 1.5 μM PLX-treated ones (**Figure 10C**) and by 116% and 146% respectively, compared to 5 μM PLX-treated cells (**Figure 10C**).

**Figure 11:**
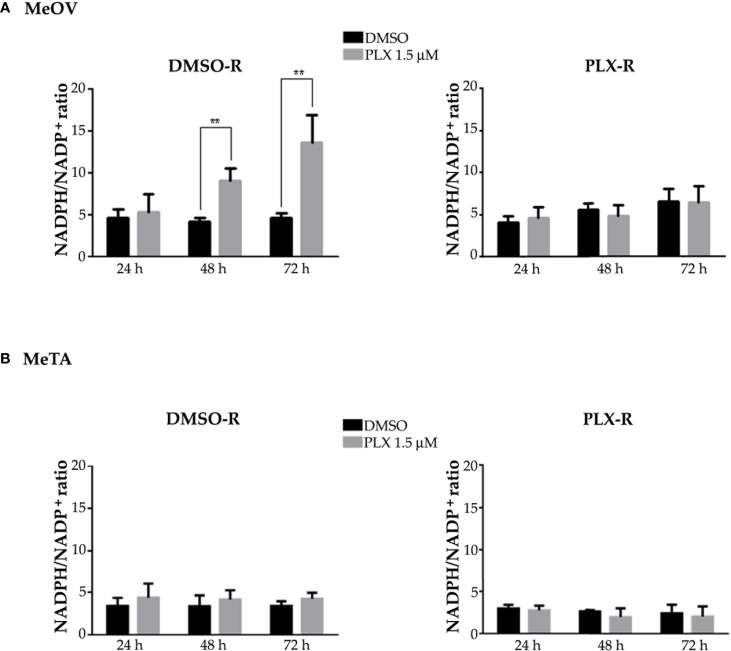
Evaluation of NADPH/NADP^+^ ratio in DMSO-R (left panels) and PLX-R (right panels) MeOV **(A)** and MeTA **(B)** melanoma cells, exposed to 1.5 µM PLX4032 or to DMSO for 24, 48 and 72 h. Histograms summarize quantitative data of the means ± S.E.M. of four independent experiments. **p<0.01 vs. untreated cells (Ctr).

Since intracellular GSH amount depends on the presence of NADPH levels, the NADPH/NADP^+^ ratio was evaluated. As shown in **Figure 11**, the NADPH/NADP^+^ ratio was increased by 2 fold and 3 fold after 48 and 72 h PLX4032 exposure, respectively, in DMSO-R MeOV cells while the ratio did not change in DMSO-R MeTa cells and in both PLX-R cell populations (**Figure 11A**, left panel).

### 3.4 PCA confirms that PLX-R cells are characterized by OXPHOS rewiring and maintenance of PDH activity and high levels of GSH

PCA was conducted on the dataset composed of 108 samples in order to interpret the results and identify the variables responsible for the acquisition of chemoresistance. The experimental plan was designed by considering all possible combinations between the factors, which include two cell lines (MeOV, MeTA), two types of drug resistance (DMSO-R, PLX-R), and three time points (24, 48, 72 h). Each combination was repeated in triplicate, resulting in a total of 108 samples. The viability variable was not included in the PCA but considered as an external response useful for the interpretation of the results.

The first two components explain 57.4% and 18.0% of the variance (75.4% in total).

**Figure 12:**
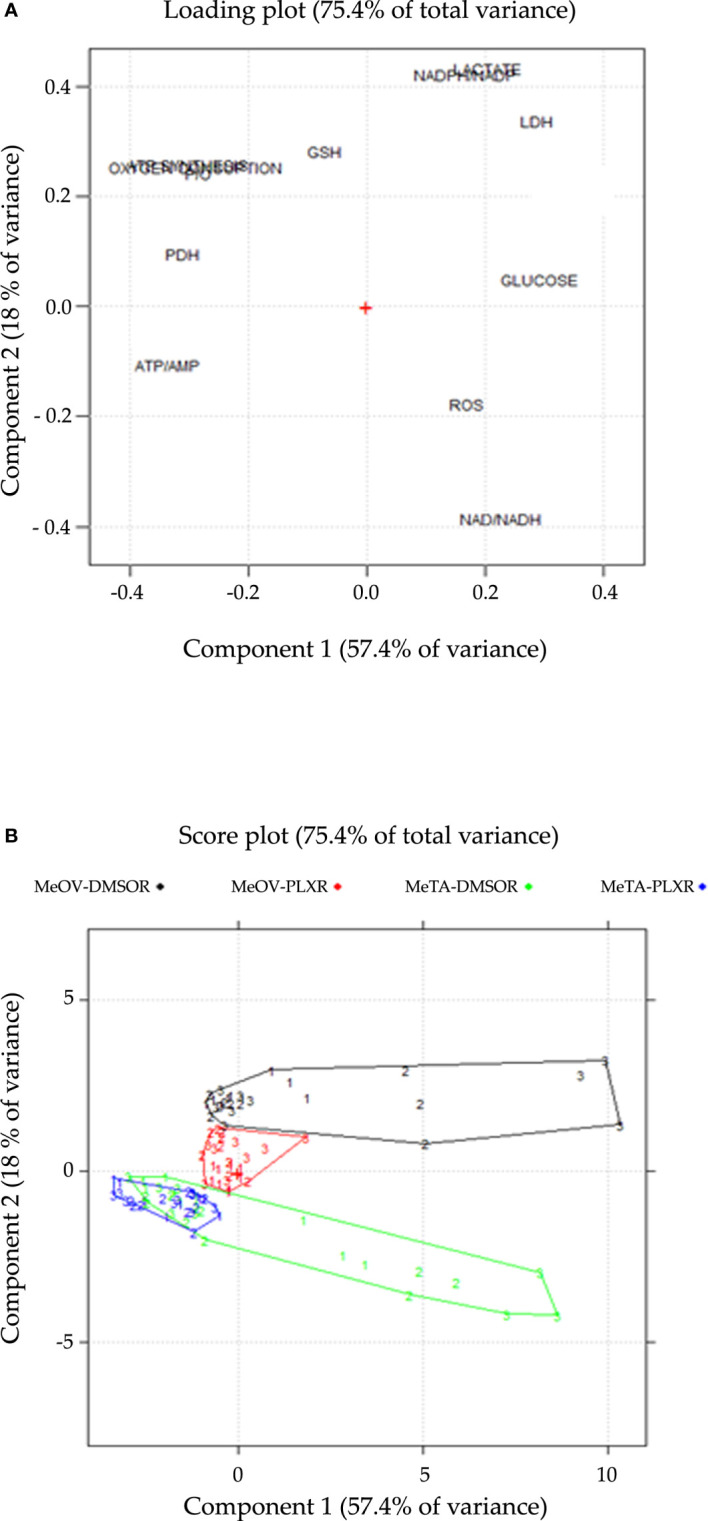
Loading **(A)** and score **(B)** plots are shown on the PC1-PC2 plane. **(A)** The loading plot describes the correlation pattern between the variables, where PC1 explains the cell viability and PC2 explains the cell-type dependent behavior in response to PLX treatment. **(B)** The score plot displays the objects in the component space, with each sample coded according to the treatment time point (1 = 24 h, 2 = 48 h, 3 = 72 h), colored and connected by a convex hull according to the cell line and the type of resistance (DMSO-R MeOV, PLX-R MeOV, DMSO-R MeTA, PLX-R MeTA).

The loading plot describes the correlation patterns of the variables (**Figure 12A**). The variables that can influence cell viability are located along Component 1 (PC1). All the parameters characterized by negative loadings (ATP/AMP, PDH activity, P/O, ATP synthesis, OCR, GSH) are associated to cell survival, while those with positive loadings (glucose consumption, LDH activity, lactate release, ROS, NAD^+^/NADH, NADPH/NADP^+^) are associated to cell death. This finding is confirmed by the strong correlation (r=-0.89; *p*<0.05) be-tween the scores on PC1 and cell viability (**Supplementary Data**). On the other hand, Component 2 (PC2) gives further information about the metabolic response of MeOV and Me-TA cells (**Figure 12A**). In this context, lactate release, NADPH/NADP^+^, LDH activity, GSH, ATP synthesis, OCR, and P/O have positive loadings, while ROS and NAD^+^/NADH have negative loadings.

The score plot (**Figure 12B**), reports all 108 samples in the significant components space, PC1-PC2 plane. The joint interpretation of loading and score plots shows that PLX-R cells acutely-treated with PLX, as well as untreated cells, have negative scores on PC1 for all time-points of treatment. In contrast, both PLX-treated DMSO-R cells exhibit a parallel and progressive increase on PC1 and more positive scores on PC2 than PLX-R cells. Moreover, as shown in the score plot (**Figure 12B**), the two MeOV populations (DMSO-R and PLX-R) are clearly separated while the MeTA populations overlap probably as a con-sequence of the specific effect of PLX4032 targeting BRAFV600E mutation which is present in MeOV and not in MeTA cells.

Therefore, PCA confirms that PLX-R melanoma cells are able to survive to drug treatment via a metabolic adaptation.

## 4 Discussion

Malignant melanoma is the most lethal form of skin cancer and although many therapeutic approaches have been developed to improve patient life expectancy, many efforts are still needed to counteract the most aggressive metastatic melanoma. In this regard, treatment guidelines establish surgical resection as the standard of care for localized and locoregional disease (3) and Vemurafenib, a BRAF inhibitor, is approved for treatment of BRAF-mutated metastatic melanoma patients (12) that has been found to induce a positive response in ~50% of patients (30). In fact, BRAF is an attractive therapeutic target for BRAF^V600E^ melanoma because this mutation has been found in a high number of patients, respect to other variants like V600K, V600D and V600R (31, 32). These mutations result in the constitutive activation of MAPK-dependent pathway thus driving the progression of melanoma (33). Although PLX4032 is a specific inhibitor of BRAF^V600E^ (34), it has been demonstrated to be active also for the V600K and V600D isoforms (35). Unfortunately, after 6-7 months of treatment with Vemurafenib, a high number of patients shows a melanoma relapse due to the acquisition of chemoresistance (13, 36). Therefore, investigating the molecular mechanisms underlying acquired PLX4032-resistance is fundamental to improve therapy efficacy and increase patients’ survival.

For this reason, several *in vitro* models of PLX4032-resistance have been proposed and studied (37), although the discrepancy between *in vitro* and *in vivo* results often limits their clinical translation. The main reason consists in the high degree of cell heterogeneity found in melanoma patients (38). In this context, the *in vitro* model realized by Delgado et al. (39) has been obtained by selecting a single-cell derived clone. Instead, the herein described cell model has been obtained by chronically treating the total cell population with the drug in order to allow the maintenance of cellular heterogeneity. The selection of PLX-resistant melanoma cells has been widely reported in the literature (40–43), but, to our knowledge, our study is the first that has been carried out by using patients’ derived metastatic melanoma cells. Therefore, in comparison with other models realized with the stabilized cancer cells (40–43), we believe that our model of acquired drug resistance is more representative of what occurs *in vivo* and, consequently, could be predictive of patient’s treatment responses.

Moreover, since in melanoma patients Vemurafenib quickly reaches a high plasma concentration and has a long half-life, the more appropriate and reliable *in vitro* strategy to mimic this condition, is represented by a continuous treatment of cancer cells with the drug (44). In this context, Fofaria et al. have used a different method to select Vemurafenib resistant cells via a pulsed treatment that does not reproduce the clinical administration of the drug (45).

Then, to better investigate the mechanisms of PLX4032 resistance, our study has been performed on two patient-derived metastatic melanoma cell lines: MeOV displaying BRAF^V600E^ mutation and MeTA displaying BRAF^V600D^ mutation.

Taking advantage of these models, here we provide strong evidence supporting the role played by the oxidative metabolism in the onset of melanoma PLX4032-resistance. Although it is already known in the literature that melanoma cells react to PLX4032-induced stress by reprogramming their energetic metabolism (10, 25, 39–41, 46–48), the mechanisms responsible for this adaptative response need to be further investigated to find new specific targets for improving the efficacy of the current therapy. In this context, glycolysis, TCA cycle and OXPHOS targeting are being tested in several clinical trials (49–52) and, based on *in vitro* and *in vivo* studies (39–41), it has been hypothesized that targeting OXPHOS with drugs, such as phenformin and metformin, could enhance the antitumor activity of BRAF inhibitors (53).

The herein results confirm that PLX4032-resistant cells are able to maintain an efficient OXPHOS metabolism. However, considering that this metabolic adaptation has been observed in both melanoma cells with different BRAF mutation and in etoposide resistant neuroblastoma cells (18), we believe that this response is neither cancer-type specific nor related to the specific drug-induced cytotoxic effect. Instead, both PLX4032-sensitive cells, differently from the resistant counterpart, exhibit i) a glycolytic profile characterized by the increase in LDH activity, in lactate release and in NAD^+^/NADH ratio and ii) OXPHOS uncoupling with enhanced intracellular AMP levels and a parallel decrease in ATP amount leading to a reduced ATP/AMP ratio. This result is in agreement with the evidence that PLX4032 exposure is able to inhibit OXPHOS and decrease ATP production (54, 55).

Moreover, the glycolytic pathway activated by acute PLX4032 treatment of drug sensitive cells is accompanied by a marked reduction in the activity of PDH, without significant changes in its protein levels. PDH is the enzyme catalyzing the decarboxylation of pyruvate into acetyl-CoA and interconnecting glycolysis and TCA cycle (56) and its decreased activity detected in sensitive cells might be responsible for shifting metabolism towards glycolysis. Conversely, in PLX-R cells PDH activity does not change whereas the protein levels increase: this data might suggest that the resistant cells become able to counteract the drug-induced inhibition of PDH activity (54) by increasing PDH expression and leading to a metabolic rewiring from glycolysis to OXPHOS that allows melanoma cells to produce sufficient ATP levels and to survive. In fact, the pre-treatment of PLX sensitive cells with DCA, a PDH activator, markedly increases the survival of PLX4032-treated cells suggesting that PDH inhibition may play a key role in sensitizing melanoma cells to PLX4032.

In parallel with mitochondrial respiration, ROS production is enhanced in resistant cells that respond with an antioxidant response by increasing GSH levels. In this regard, it has been demonstrated that slow-cycling melanoma cells intrinsically resistant to PLX4032, to many chemotherapeutic drugs and to radiotherapy, overexpressed KDM5B, the histone H3KA demethylase which contribute to maintain OXPHOS and to enhance GSH-related processes (57–60). Although the metabolic adaptations observed in our model are similar to those reported in KDM5B-overexpressing cells, the mechanism of resistance is different: the first case represents an adaptative response of the whole cell population exposed to PLX4032 for six months; the second one is an intrinsic resistance of a small sub-population. In addition, another discrepancy between these two models of resistance is that PLX-R cells do not show significant changes in cell cycle phases distribution, whereas KDM5B cells are slow-cycling. Therefore, although the metabolic adaptations observed in intrinsic and acquired resistance are similar, it is reasonable to hypothesize that the molecule players involved in the adaptative response may be different.

Our data agree with studies reporting that PDH activity is important to maintain redox homeostasis and to support GSH regeneration, via the consumption of NADPH which originates by the activation of the pentose phosphate pathway (PPP) (61–63). In this context, Khamari el al (64)., by using mice model and melanoma cells generated from mice tumors, have demonstrated that PPP is induced by activation of NRF2, the main transcription factor regulating antioxidant response and GSH biosynthesis.

GSH and redox homeostasis have been widely demonstrated to play a crucial role in therapy-resistance (18, 65–67) and it has been recently reported that compounds able to inhibit GSH peroxidase 4 (GPX4) induce ferroptosis in BRAF^V600E^ mutant melanoma cells increasing the sensitivity to Vemurafenib (68). Interestingly, the pre-treatment with NAC, a GSH synthesis precursor, is able to reduce the cytotoxic effect of PLX4032 in both sensitive cell populations. This data confirms the crucial role of GSH in sensitizing melanoma cells to the drug in agreement to other studies carried out in several human cancers treated with traditional therapies (18, 69–71). In conclusion, based on the results obtained in this innovative model of acquired PLX4032 resistance, it is possible to believe that among the factors underlying resistance, a crucial role is played by the maintenance of PDH activity and by the increase in intracellular GSH levels. Therefore, although further studies are needed, it is possible to hypothesize that inhibitors of PDH activity and/or of GSH biosynthesis could be used in combination with PLX4032 to overcome drug resistance of BRAF-mutated melanoma patients.

## Data availability statement

The original contributions presented in the study are included in the article/**Supplementary Material**. Further inquiries can be directed to the corresponding author.

## Author contributions

BM and CD contributed to conception and design of the study. OG, GEV, LM, SR, SB, AB, EF, MG, SV, and BM performed experiments. OG, GEV, BM and CD contributed to acquisition, analyses and interpretation of data. BM, CD, GP, MCM and RL contributed to validation of data. OG, GEV, and BM wrote the first draft of the manuscript. All authors contributed to manuscript revision, read, and approved the submitted version.
