# Supplementary material for: PLX4032 resistance of patient-derived melanoma cells: crucial role of oxidative metabolism
Source: Front Oncol. 2023 Jul 18;13:1210130. doi: 10.3389/fonc.2023.1210130 (PMC10391174; doi:10.3389/fonc.2023.1210130)
Supplement: Supplementary file 1 [file DataSheet_1.docx]

Supplementary Material

PLX4032 resistance of patient-derived melanoma cells: crucial role of oxidative metabolism

**Ombretta Garbarino^1✝^, Giulia Elda Valenti^1✝^, Lorenzo Monteleone^1^, Gabriella Pietra^1,2^, Mingari Maria Cristina^1,2^, Andrea Benzi^3^, Santina Bruzzone^2,3^, Silvia Ravera^4^, Riccardo Leardi^5^, Emanuele Farinini^5^, Stefania Vernazza^1^, Melania Grottoli^1^, Barbara Marengo^1✝^ and Cinzia Domenicotti^1*✝^**

**✝** These authors contributed equally to this work

*** Correspondence:**Cinzia Domenicotti[cinzia.domenicotti@unige.it](mailto:cinzia.domenicotti@unige.it)


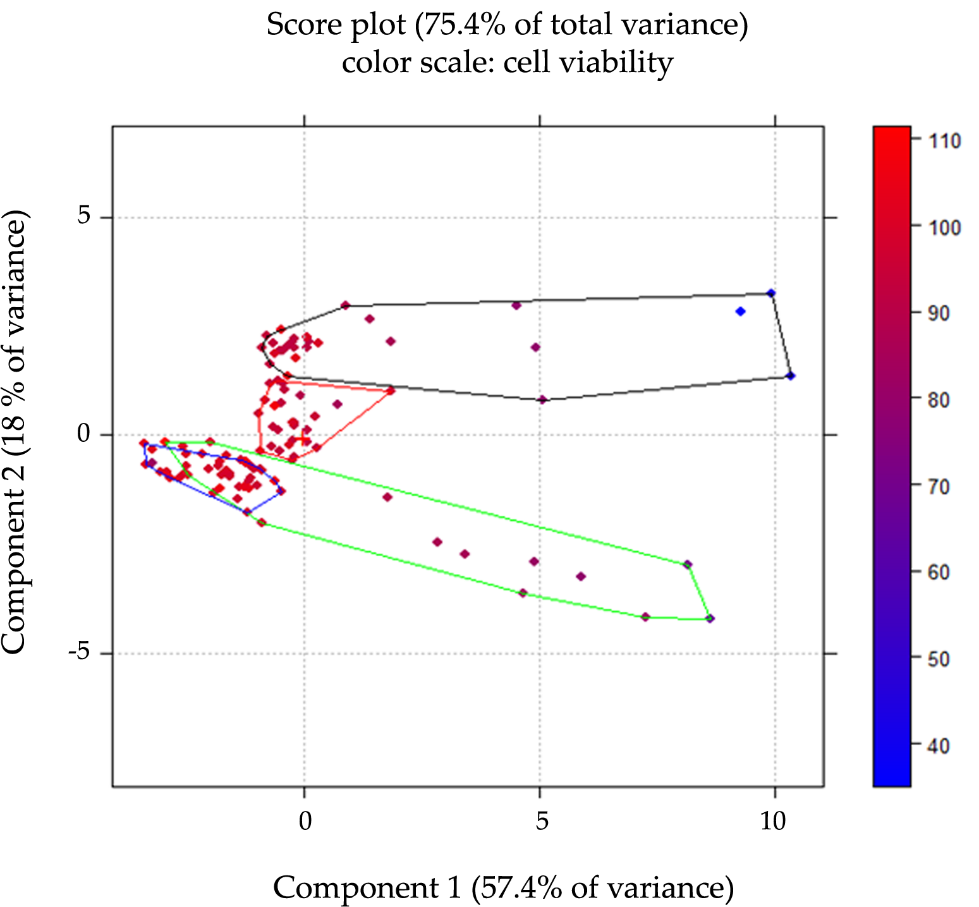


**Supplementary Figure 1.** Supplementary data. The score plot displays the objects in the component space, with each sample connected by a convex hull according to the cell line and the type of resistance (MeOV-DMSO-R, MeOV-PLX-R, MeTA-DMSO-R, MeTA-PLX-R) and colored according to the independently measured viability variable (r=-0.89; p<0.05).
