# Supplementary material for: PLX4032 resistance of patient-derived melanoma cells: crucial role of oxidative metabolism
Source: Front Oncol. 2023 Jul 18;13:1210130. doi: 10.3389/fonc.2023.1210130 (PMC10391174; doi:10.3389/fonc.2023.1210130)
Supplement: Supplementary file 2 [file Table_1.docx]

Supplementary Material

PLX4032 resistance of patient-derived melanoma cells: crucial role of oxidative metabolism

**Ombretta Garbarino^1✝^, Giulia Elda Valenti^1✝^, Lorenzo Monteleone^1^, Gabriella Pietra^1,2^, Mingari Maria Cristina^1,2^, Andrea Benzi^3^, Santina Bruzzone^2,3^, Silvia Ravera^4^, Riccardo Leardi^5^, Emanuele Farinini^5^, Stefania Vernazza^1^, Melania Grottoli^1^, Barbara Marengo^1✝^ and Cinzia Domenicotti^1*✝^**

*** Correspondence:** Cinzia Domenicotti [cinzia.domenicotti@unige.it](mailto:cinzia.domenicotti@unige.it)

**Supplementary Table 1.** **ATP and AMP intracellular levels.** The intracellular levels of ATP and AMP were evaluated in DMSO- and PLX-R melanoma cells exposed to 1.5 µM PLX4032 (PLX) or to DMSO for 24, 48 and 72 h.

|  | **MeOV** | | **MeTA** | |
| --- | --- | --- | --- | --- |
|  | **ATP** | **AMP** | **ATP** | **AMP** |
| **DMSO-R untreated (24 h)** | 1.08±0.02 | 0.96±0.03 | 1.3±0.12 | 0.8±0.03 |
| **DMSO-R + DMSO (24 h)** | 1.07±0.05 | 0.93±0.03 | 1.29±0.13 | 0.8±0.03 |
| **DMSO-R + 1.5 μM PLX (24 h)** | 0.94±0.04**^^^^** | 1.07±0.06 | 0.91±0.08**^^^^** | 0.96±0.03**^^^^** |
| **DMSO-R untreated (48 h)** | 1.07±0.03 | 0.93±0.03 | 1.3±0.14 | 0.8±0.04 |
| **DMSO-R + DMSO (48 h)** | 1.08±0.01 | 0.95±0.02 | 1.3±0.1 | 0.81±0.03 |
| **DMSO-R + 1.5 μM PLX (48 h)** | 0.83±0.06^***^ | 1.23±0.03**^****^** | 0.73±0.04**^***^** | 1.04±0.05**^***^** |
| **DMSO-R untreated (72 h)** | 1.02±0.06 | 0.94±0.04 | 1.29±0.11 | 0.81±0.03 |
| **DMSO-R + DMSO (72 h)** | 1.06±0.05 | 0.95±0.05 | 1.29±0.12 | 0.81±0.02 |
| **DMSO-R + 1.5 μM PLX (72 h)** | 0.45±0.04**^••••^** | 1.39±0.03**^••••^** | 0.56±0.03**^•••^** | 1.08±0.07**^•••^** |
| **PLX-R untreated (24 h)** | 1.14±0.06 | 0.91±0.02 | 1.15±0.03 | 0.83±0.04 |
| **PLX-R + DMSO (24 h)** | 1.13±0.04 | 0.92±0.03 | 1.16±0.05 | 0.81±0.06 |
| **PLX-R + 1.5 μM PLX (24 h)** | 1.13±0.03 | 0.93±0.04 | 1.14±0.04 | 0.81±0.04 |
| **PLX-R untreated (48 h)** | 1.16±0.09 | 0.92±0.02 | 1.19±0.06 | 0.82±0.03 |
| **PLX-R + DMSO (48 h)** | 1.15±0.07 | 0.92±0.04 | 1.18±0.02 | 0.82±0.05 |
| **PLX-R + 1.5 μM PLX (48 h)** | 1.14±0.07 | 0.94±0.05 | 1.17±0.04 | 0.81±0.01 |
| **PLX-R untreated (72 h)** | 1.11±0.04 | 0.92±0.06 | 1.17±0.05 | 0.81±0.02 |
| **PLX-R + DMSO (72 h)** | 1.14±0.04 | 0.95±0.04 | 1.18±0.07 | 0.82±0.03 |
| **PLX-R + 1.5 mM PLX (72 h)** | 1.02±0.06 | 1.05±0.03**^•^** | 1.18±0.06 | 0.8±0.04 |

^^*p*<0.01 vs untreated DMSO-R 24 h; ****p*<0.001 vs untreated DMSO-R 48 h; *****p*<0.0001 vs untreated DMSO-R 48 h; ^•^*p*<0.05 vs untreated DMSO-R 72 h; ^•••^*p*<0.001 vs untreated DMSO-R 72 h; ^••••^*p*<0.0001 vs untreated DMSO-R 72 h
